# “Here in the Bible Belt, It’s Predominantly Negative”: Sexual Identity Stigma in the American South, 50 Years After Stonewall

**DOI:** 10.3389/fpsyg.2021.804064

**Published:** 2021-12-21

**Authors:** Joseph J. Frey, William J. Hall, Jeremy T. Goldbach, Paul Lanier

**Affiliations:** ^1^Department of Social Work, University of North Texas, Denton, TX, United States; ^2^School of Social Work, University of North Carolina at Chapel Hill, Chapel Hill, NC, United States; ^3^The Brown School, Washington University in St. Louis, St. Louis, MO, United States

**Keywords:** LGBTQ, sexual orientation, sexual identity, stigma, minority stress, United States South, qualitative

## Abstract

Lesbian, gay, bisexual, and pansexual (LGB+) individuals have disproportionate rates of mental illness. Minority stress and sexual identity stigma are posited as the primary social determinants of LGB+ mental health disparities. Discussions in the literature have questioned the impact of sexual identity stigma in a world increasingly accepting of sexual minorities. Additionally, the LGB+ population in the United States South is often overlooked in American research. This article details a qualitative study exploring experiences related to sexual identity stigma among adults who identify as LGB+ in the United States South. Semi-structured interviews with 16 individuals were analyzed using content analysis. Six thematic categories of stigma emerged from participants’ experiences: (a) navigating an LGB+ identity, (b) social acceptability of an LGB+ identity, (c) expectation of LGB+ stigma, (d) interpersonal discrimination and harassment, (e) structural stigma, and (f) relationship with the lesbian, gay, bisexual, transgender, and queer (LGBTQ) community. Findings suggest that sexual identity stigma remains a common experience among these Southern United States participants. Further, thematic categories and subcategories primarily aligned with extant theory with one exception: Intracommunity stigma, a form of stigma emanating from the LGBTQ community, emerged as a stigma type not currently accounted for in theoretical foundations underpinning mental health disparities in this population.

## Introduction

Adults who identify as lesbian, gay, bisexual, and pansexual (LGB+) are disproportionately burdened by mental illness (Bostwick et al., 2010; Institute of Medicine, 2011). Research indicates that 37% of LGB+ adults experienced any mental illness in the past year, more than double that reported by heterosexuals (Medley et al., 2016). Further, LGB+ individuals have at least 1.5 times higher risk of depression and anxiety disorders and at least 2 times higher risk of suicide attempt, with some studies finding that up to 20% of LGB+ individuals attempt suicide in their lifetime (King et al., 2008; Hottes et al., 2016). Rooted in the stigma scholarship of Goffman (1963), Brooks (1981), and Link and Phelan (2001), minority stress theory (Meyer, 2003) and the sexual stigma framework (Herek, 2007) are the primary explanatory frameworks used to understand mental health disparities in the LGB+ population.

It has typically been accepted that the United States LGB+ population experiences pervasive sexual identity stigma. As defined by Herek (2007), sexual identity stigma is the “negative regard, inferior status, and relative powerlessness that society collectively accords to any nonheterosexual behavior, identity, relationship, or community” (p. 906). Societal responses to human difference that lead to stigmatized identities can foster status loss, stereotyping, and discrimination (Link and Phelan, 2001). Positive shifts in the sociopolitical environment for LGB+ individuals in the United States, as indicated by increasing societal acceptance and advancement of civil rights (e.g., marriage equality), have been cause for celebration by champions of LGB+ rights. With this progress, some now believe that stigma is a negligible factor in the lives of LGB+ people. Moreover, there is evidence of varying levels of LGB+ acceptance across the United States (Loftus, 2001; Pew Research Center, 2013). Indeed, scholarship exploring differences in experiences by geographic location (e.g., rural vs. urban, regional, and state boundaries) for individuals with LGB+ identities is present in the extant literature, yet the experiences of LGB+ people in the American South continue to be neglected in existing research. Given the overall positive shifts in the American sociopolitical climate, questions about present levels of sexual identity stigma, and lack of research regarding LGB+ experiences in the United States South, the present study explored stigmatization of LGB+ identities.

### Stigma and LGB+ Identities

An LGB+ identity is a social identity with associated stigma. Scholarly exploration of stigma began with the early work of Goffman (1963), who first defined stigma as “an attribute that is deeply discrediting within a particular social interaction” (p. 3). An expanded definition of stigma from Link and Phelan (2001) includes the negative stereotyping of differences leading to unequal treatment and disparate life outcomes. And although the efforts of Goffman (1963) spurred stigma-focused scholarship, application of the stigma construct to LGB+ lives was slow to emerge. Due to both stigmatization of LGB+ identities and fear of further pathologizing this population, the delayed advent of LGB+ stigma research affected our understanding of stigma in LGB+ lives. The scholarship of Brooks (1981) labeled stigma’s effects on lesbians as “minority stress,” creating a foundation for later exploration of sexual identity stigma. Grounded in this earlier work, the minority stress theory of Meyer (2003) seeks to explain existing LGB+ mental health disparities *via* minority stress processes at distal and proximal levels. The sexual stigma framework of Herek (2007) builds on minority stress theory and frames sexual stigma in structural and individual manifestations.

Both sexual stigma and minority stress are conceptualized in a heterosexist society that perpetuates the norm of heterosexuality and the inferiority of LGB+ people (Meyer, 1995; Herek, 2007). Minority stress theory describes distal stressors in the environment as prejudicial events, discrimination, and violence directed at LGB+ people (Meyer, 2003). Proximal stressors identified by minority stress theory include sexual identity concealment (i.e., hiding an LGB+ identity either as a form of protection from stigmatization or due to feelings of guilt or shame); expectations of rejection from others due to an LGB+ identity, which may result in heightened vigilance as a form of protection; and internalized homophobia (i.e., internalization of society’s negative attitudes toward LGB+ identities; Meyer, 2003).

The sexual stigma framework (Herek, 2007) describes structural stigma as the laws, policies, and statements of heterosexist institutions that perpetuate the norm of heterosexuality and the inferiority of LGB+ people. This inferiority fosters the invisibility of LGB+ individuals and the subsequent application of the “abnormal” label to sexual minorities. The individual manifestations of sexual stigma include enacted stigma (i.e., discriminatory acts, prejudicial behavioral expression, and violence); felt stigma or expected stigma (i.e., expectation of negative stigmatization from a heterosexist society and any vigilance employed for protective purposes); and internalized stigma (i.e., adoption and self-application of society’s negative attitudes toward LGB+ people; Herek, 2007).

Empirical examination of structural stigma (Hatzenbuehler et al., 2009, 2012), discrimination or enacted stigma (Meyer, 1995, 2003; Lehavot and Simoni, 2011), expectations of rejection or stigma (Meyer, 1995, 2003), and internalized stigma and identity concealment (Meyer, 1995, 2003; Lehavot and Simoni, 2011) revealed the significance of these constructs in LGB+ lives. Extant research demonstrated a strong link between sexual identity stigmatization, the resulting stress, and negative mental health outcomes in the LGB+ population (e.g., Meyer, 1995, 2003, 2007; Hatzenbuehler et al., 2008; Lehavot and Simoni, 2011).

### The Evolving American Sociopolitical Landscape for Lesbians, Gays, and Bisexuals

Stigmatization of LGB+ identities in the United States is primarily attributed to conservative social, political, and religious beliefs that prescribe “traditional values” (Bean and Martinez, 2014; Schnabel, 2016; Costa et al., 2019). Linked religious doctrine and political beliefs have furthered sexual minority stigmatization (Schnabel, 2016) and historically, theological beliefs frequently precluded acceptance of people who identified as LGB+, constructing them as immoral, criminal, and mentally ill (Boswell, 1980; Herek et al., 2007; Rosati et al., 2020).

In 2019, the lesbian, gay, bisexual, transgender, and queer (LGBTQ) community and its allies marked the 50th anniversary of the Stonewall Riots. Since Stonewall, social acceptance of people who identify as lesbian and gay has increased in the United States. Examination of nationally representative General Social Survey data revealed that Americans’ attitudes about same-sex sexual relations stagnated from 1973 through the early 1990s, with a favorable trend beginning at that time (Loftus, 2001; Schnabel, 2016). Although views on same-sex relations remain polarized, the number of Americans who indicated same-sex sexual relations was not wrong at all improved from 12% in 1987 to 49% in 2014 (Associated Press-NORC Center for Public Affairs Research, 2015). Polling by Gallup indicated similar growth in Americans’ acceptance of marriage between same-sex couples, finding support for its legal validity has gradually risen from 27% in 1996 to 64% in 2017 (McCarthy, 2017). This progression of attitudinal shifts in the United States concerning lesbian and gay identities represents a dramatic change during the past 50+ years.

Gains in social acceptance of lesbian and gay individuals contribute to and exist alongside advances in LGB+ civil rights. In the decades preceding marriage equality granted by the landmark 2015 Supreme Court decision Obergefell v. Hodges, the LGBTQ movement experienced both successes and setbacks. Beyond marriage equality, advancements in LGB+ civil rights include legal permissibility of private, same-sex sexual behavior between consenting adults secured by the 2003 Supreme Court decision in Lawrence v. Texas and the 2011 repeal of the “Don’t Ask, Don’t Tell” policy that ended the ban on openly LGB+ individuals serving in the United States military (Frank, 2013). Marriage equality is viewed by many as the crowning achievement of the LGBTQ civil rights movement in the United States, but the fight for civil marriage between same-sex couples in the United States was an ongoing battle beginning in 1971 (Aminian et al., 2017). Though securing the right to marry was an important milestone in the fight for LGB+ equality, battles continue to be waged to protect the rights and lives of LGB+ people.

### Questioning of Stigma Levels

With partial success in securing LGB+ rights and gains in social acceptance, the question arises of whether sexual identity stigma remains a significant force in LGB+ lives. Meyer (2016), citing advances in civil rights, stated that “it is reasonable to ask whether minority stress is still relevant to the study of LGBT health” (p. 84). Further responding to this question, scholars, such as Savin-Williams (2005) and McCormack (2012), have, in certain instances, declared the era of sexual identity stigma to be a thing of the past. Such sentiments have reached the popular media as well, wherein the fight for “gay rights” has been declared “over” (Kirchick, 2019). Indeed, the American populace holds similar beliefs; a majority (56%) is now satisfied with social acceptance levels of lesbians and gay men (McCarthy, 2018). Though the number of scholars and writers who profess sexual identity stigma to be insignificant in our present milieu is limited, that the question has entered the lexicon of LGB+ literature is cause for consideration.

### Lesbians, Gays, and Bisexuals and the Southern United States Context

Amid an improving national landscape for the LGB+ population is the reality that not all LGB+ people in the United States share equally in gains of social acceptance and advancement of civil rights. Though social conservatism and religiosity are not unique to the United States South (or the United States), the South is often defined by these characteristics (Wilson, 2000; Valentino and Sears, 2005). Thus, the South has typically been deemed a less hospitable climate for individuals identifying as LGB+. However, the South includes the largest population of LGBTQ adults (35%) in the United States (Hasenbush et al., 2014), including nearly 3.5 million LGB+ adults (Williams Institute, 2019). As defined by the United States Census Bureau (U.S. Department of Commerce, 1994), 16 states and the nation’s capital make up the Southern states and include Alabama, Arkansas, Delaware, Florida, Georgia, Kentucky, Louisiana, Maryland, Mississippi, North Carolina, Oklahoma, South Carolina, Tennessee, Texas, Virginia, West Virginia, and the District of Columbia.

Although the American South is home to the largest proportion of the country’s LGB+ population, regional differences in structural stigmatization experienced by LGB+ people there are evident. LGB+ people in the South are less likely to be protected from sexual orientation-based discrimination compared to their peers elsewhere (Hasenbush et al., 2014; Cramer et al., 2017). When compared to states in the Midwest, Northeast, and West, the South has the fewest states by percentage that prohibit sexual orientation discrimination in employment practices, housing practices, and public accommodations; the only Southern states providing such protections are Delaware, Maryland, Virginia, and Washington, DC (Mallory and Sears, 2015; Cramer et al., 2017; Hammer et al., 2020)—none of which are part of the “Deep South” (i.e., Alabama, Georgia, Louisiana, Mississippi, North Carolina, and South Carolina). Currently, limited statewide protections are available in Florida, Kentucky, North Carolina, and Texas, which prohibit discrimination based on sexual orientation for state government employees (Movement Advancement Project, 2021). Among Southern states lacking nondiscrimination laws protective of the LGB+ population, some communities have enacted such laws at the local level; nationwide, the only states that ban cities and counties from enacting or enforcing nondiscrimination laws are in the South—Arkansas and Tennessee (Movement Advancement Project, 2021).

In addition to exclusionary laws, anti-LGB+ laws in the South add further stigmatization. Such anti-LGB+ laws have included same-sex marriage bans that existed prior to marriage equality (in all Southern states except for Delaware, Louisiana, Maryland, and Washington, DC; Aminian et al., 2017) and infamous “bathroom bills.” For example, in 2016, North Carolina passed the Public Facilities Privacy and Security Act (i.e., HB 2) that required individuals use public facility bathrooms in alignment with the sex listed on their birth certificate; further, this law blocked local anti-discrimination ordinances, thus nullifying existing local LGBTQ protections in communities across North Carolina (Fausset, 2017). The replacement bill to address HB 2 eliminated the public facility bathroom requirement in 2017 and allowed local nondiscrimination ordinances beginning in 2020 (Fausset, 2017). Moreover, structural stigmatization of sexual minorities is notable in state sex education policies; a recent study found explicit stigmatization of homosexuality (i.e., homosexuality described as a lifestyle choice, unacceptable, unhealthy, or criminal) in the policies of eight states, six of which are in the South (i.e., five Deep South states of Alabama, Louisiana, Mississippi, North Carolina, and South Carolina, along with Oklahoma; Hall et al., 2019), further differentiating the region’s level of sexual identity stigmatization from other United States regions.

Marginalization of Southern LGB+ individuals also occurs in research. Southern LGB+ populations are frequently overlooked for study in favor of populations in major American cities where large populations of LGB+ people reside, typically in the North and West regions of the nation (Baunach and Burgess, 2013; Irwin and Austin, 2013; Hall, 2018). However, to understand and address health disparities among LGB+ populations, increased research inclusive of Southern LGB+ lives is needed. When geography is considered, health disparities among Southern LGB+ people are the greatest in the nation (Austin and Irwin, 2010). Examples of recent research focused on the lives of LGB+ Southerners include a study by Austin (2013) on sexual identity disclosure to healthcare providers by Southern lesbians, a study by Irwin and Austin (2013) exploring suicidal ideation among Southern White lesbians, and a study by Griffin et al. (2019) examining anxiety among sexual minorities in the South. However, these studies, and those similar in purpose, did little to further understanding of sexual identity stigma experienced by the Southern LGB+ population.

The limited existing research indicates LGB+ people in the South experience greater discrimination due to their sexual orientation. Findings from GLSEN’s National School Climate Survey, a national survey of 23,001 LGBTQ middle and high school students regarding their school experiences, indicate that Southern LGBTQ students experience a more hostile school climate compared to their peers in other United States regions; for example, 76.3% reported experiencing victimization (i.e., bullying, harassment, and assault) due to sexual orientation, compared to 73.1% in the Midwest, 67.4% in the West, and 64.3% in the Northeast (Kosciw et al., 2018). For many sexual minorities, this type of discrimination continues into adulthood. In a nationally representative survey of 1,197 LGBTQ adults, 66% indicated they experienced LGBTQ-related discrimination (i.e., subject to slurs or jokes, rejected by a family member or friend, threatened or physically attacked, made to feel unwelcome at a place of worship, received poor service in a business establishment, and treated unfairly by an employer); 29% of LGBTQ adults in the South experienced four of more forms of discrimination compared to 22% in the West, 19% in the Midwest, and 18% in the Northeast (Pew Research Center, 2013). No recent qualitative studies were identified in the extant literature comprehensively exploring how LGB+ Southerners experience sexual identity stigma.

### Current Study

Stigmatization of LGB+ identities is prevalent in American society, with regional differences. Scant research on sexual identity stigma experienced by LGB+ Southerners has emerged, creating an evidence gap. Sexual identity stigma scholarship has proliferated in the past decade due in part to recognition of LGB+ mental health disparities as a public health concern by multiple entities, including the Institute of Medicine’s (now the National Academy of Medicine) 2011 report on LGBTQ health and Healthy People 2020 and 2030. Improving socio-environmental conditions (e.g., marriage equality and increased societal acceptance) for people with LGB+ identities is cited as evidence of decreasing levels of sexual identity stigmatization. If indeed sexual identity stigmatization is decreasing, this would have important implications for its future study and for research of minority stress and LGB+ health disparities. With changes in social acceptance of LGB+ people and gains in achieving civil rights, the experiences of Southern LGB+ people are often not considered or absent from the scholarship. Using an exploratory qualitative approach, this study examined the question: What are the experiences related to sexual identity stigma among self-identified LGB+ adults in the present-day United States South?

## Materials and Methods

### Participants

Interviews were conducted between November 2018 and February 2019 with a purposive sample of 16 self-identified LGB+ adults in a suburban and urban region of North Carolina. Adequacy of the sample size was determined through assessment of data saturation—the process of examining when to stop data collection based on the ending of new code and theme development (Padgett, 2008; Schreier, 2014). North Carolina was selected as the study site because it is an exemplar Southern state. North Carolina, similar to the majority of its Southern contemporaries, lacks statewide LGB+ protections (e.g., housing, employment, and public accommodations) and has a sociopolitical history of LGB+ stigmatization. To be eligible to participate, individuals had to: (a) self-identify as LGB+; (b) be 18 years old or older; and (c) be fluent in English. Participants were recruited for the current study *via* flyers posted at three community-based organizations—two LGBTQ centers and one Latinx-serving agency that provided LGBTQ-specific services. Additionally, recruitment materials were circulated *via* LGBTQ-focused listservs. The Institutional Review Board of the University of North Carolina at Chapel Hill approved all study procedures.

The 16 study participants self-identified as lesbian (*n* = 5), gay (*n* = 5), bisexual (*n* = 4), or pansexual (*n* = 2) and ranged in age from 18 to 50 years (*M* = 30.4, SD = 11.4). Participants’ racial and ethnic identities were White (*n* = 10), Asian (*n* = 2), biracial (*n* = 2), Black (*n* = 1), and Latinx (*n* = 1); participants identified as women (*n* = 9), men (*n* = 6), and gender nonbinary (*n* = 1). The range of educational attainment among participants included a high school diploma (*n* = 1), some college (*n* = 5), an associate degree (*n* = 2), a bachelor’s degree (*n* = 4), and a master’s degree (*n* = 4). All participants resided in North Carolina when interviewed. However, the length of time participants resided in the South and in North Carolina varied, with both ranging from 1.25 to 50 years; mean length of residence in the South was 25.1 years (SD = 13.1) and mean length of residence in North Carolina was 18.6 years (SD = 13.9). Participants were raised (primary location) in various locales: suburban or urban North Carolina (*n* = 7), rural North Carolina (*n* = 1), a different Southern state (*n* = 5), a non-Southern state (*n* = 1), and outside of the United States (*n* = 2).

### Data Collection Procedures

Qualitative data were collected through in-depth semi-structured interviews conducted by the first author. The times and locations of all interviews were based on participant availability and convenience. Participants were offered three options for interview location, all of which provided privacy (i.e., university office, reserved room in a public or university library, or reserved room in a coworking space). Additionally, participants had the opportunity to suggest an alternative interview location that may offer greater convenience or comfort. Participants received a $20 gift card in appreciation of their time. Immediately preceding each interview, the author obtained written informed consent to conduct and digitally audio record the interview.

The interviews were conducted using a semi-structured interview guide consisting of open-ended questions and follow-up probes developed by the first author. Though existing theories (e.g., sexual stigma framework by Herek, 2007; minority stress theory by Meyer, 2003) provided a backdrop for the current study, this interview guide was developed using an exploratory lens to allow Southern LGB+ experiences to emerge. Interview topics (with example questions and prompts) fostered exploration of participants’: (a) demographic characteristics (how do you identify your sexual orientation? how would you describe your level of outness?); (b) perceptions of the LGB+ friendliness of their environment (what makes a community LGB+ friendly? thinking about the community where you live, describe its level of LGB+ friendliness.); (c) coming out experiences (tell me about your coming out experiences, what was it like to come out to your family?); (d) connection to the LGBTQ community (how strongly do you identify with the LGBTQ community?); (e) experiences with sexual identity stigma (how do you view your LGB+ identity? describe LGB+ related attitudes in your family. what are your experiences with LGB+ related discrimination? what are your thoughts on the LGBTQ community?); and (f) thoughts on LGB+ laws and policies (what LGB+ related laws are you aware of? how do these laws affect you?). Mean interview length was approximately 1 h. Audio files of interviews were transcribed verbatim by the first author and deidentified to promote participant confidentiality.

### Analytic Strategy

Data were analyzed using an inductive approach to qualitative content analysis that allowed for both explications of meaning and the emergence of themes from the interview transcripts (Thomas, 2006; Schreier, 2012; Cho and Lee, 2014). Analysis of data involved three steps as outlined by Schreier (2014). First, using reduced data relevant to the study’s research question, the first author open-coded eight transcripts inclusive of lesbian, gay, bisexual, and pansexual participants, and created an initial coding frame. Analytic memos were used in the coding process to track coding decisions and issues that required further analysis (Padgett, 2008). Second, the coding frame was piloted with the remaining eight transcripts, resulting in multiple modifications. After this step, the coding frame consisted of six categories and 17 subcategories. Third, all reduced transcripts were reviewed and coded by the first author using the finalized coding frame. Data analyses were conducted using QSR NVivo (version 12 for Mac).

To enhance study rigor, the first author received coding and analysis feedback from other authors through collaborative discussions regarding category and subcategory development (Padgett, 2008). Further, rigor was established by (a) creation of an audit trail consisting of deidentified interview transcripts, the lead author’s field notes written during interviews, and memos documenting coding challenges, questions, and decisions (Padgett, 2008); (b) presentation of representative quotations (Cho and Lee, 2014); and (c) substantive expert review of concept-driven categories in the coding frame to assess validity (Schreier, 2014).

## Findings

Though participants’ perspectives varied regarding the level of sexual identity stigma experienced by LGB+ people, inclusive of stigma they personally experienced and in the LGB+ population in general, each recounted occurrences of stigmatization in various forms. Six overarching thematic categories (with 17 subcategories) of stigma experienced by participants emerged from the data: (a) navigating an LGB+ identity (identity concealment; self-censorship; coming out concerns; coming out challenges; internalization of sexual identity messages; positive coming out experiences; and positive sexual identity development); (b) social acceptability of an LGB+ identity (faith and geography; pessimism about social acceptance; and optimism about social acceptance); (c) expectation of LGB+ stigma; (d) interpersonal discrimination and harassment (personal experiences; questioning experiences as discriminatory); (e) structural stigma (feeling excluded, marginalized, or not protected; precarious nature of protections; and feelings of inclusion or protection); and (f) relationship with the LGBTQ community (conflicted relationship with the community; positive relationship with the community). Each thematic category is reviewed through interview quotes accompanied by the participant’s pseudonym, age, sexual orientation, and racial and ethnic and gender identities.

### Category 1: Navigating an LGB+ Identity

The category of navigating an LGB+ identity was characterized by participants’ coming out experiences, being out, and feelings and behaviors related to their LGB+ identity. All 16 participants discussed how they navigated a sexual minority identity; most emphasized associated challenges, whereas some also focused on positive aspects of navigating an LGB+ identity.

#### Identity Concealment

The most common challenge, highlighted by 10 participants (63%), was identity concealment. Phoebe (18, bisexual, White woman), who is not out to her family, described a particularly stressful experience she had at home:

*There was a close call a few years ago where there was a post about a friend potentially coming out to me as gay or lesbian and my dad just lost it because he like misheard, misinterpreted it, and he read it as me coming out back in a time where I wasn’t fully set in my identity, but I had a strong feeling. But just his very large, very negative reaction, just, I was terrified because he was like, “You need to get out of this house now.” And I’m just like, “What is happening?” And so just after that, I have just completely never even brought up that topic in any discussion. And in the meantime, I think about coming out to my parents, which is kind of like, I feel my heart rate go up, and I just, I immediately think, “No. Not anytime soon or even never.”*

Although Phoebe was out in limited contexts that she deemed safe, being out about her bisexuality to her family living in rural North Carolina was clearly not one of those contexts. Robin (50, lesbian, White woman) also discussed concealing sexual orientation in certain contexts as she described the identity concealment of friends who do not live in LGB+-friendly cities in North Carolina:

*I have a lot of friends who live out in [suburban or rural] areas … and they’re totally out when they come to town and then they’re totally not when they live at home*.

Likewise, Jordan (25, pansexual, White woman) described concealing her sexual orientation in specific contexts—namely, working in healthcare—as she described both her experience and the experiences of friends with being out in the workplace:

*I have never felt like I was able to be out to an employer. I feel the same way about any of my friends who identify as such, unless they were employed by an independent, progressive-thinking business owner. So, I think that kind of speaks to what's considered professional in terms of your daily life and being in your identity*.

#### Self-Censorship

In addition to concealing sexual orientation, nine participants (56%) described acts of self-censorship where they either edited their behavior or were encouraged to do so to seem “less LGB+.” For many, censoring their LGB+ identity was an act of self-preservation, as in the case of Javiera (26, bisexual, Latinx woman), who stated:

*I mean, there are definitely places where it's scary to walk down the street with someone, but anyway, it's been scary to walk down the street with my girlfriend— we’ll start with that one—and you know I've chosen not to, and we've selectively chosen not to*.

Casey (20, lesbian, biracial woman) received encouragement to censor herself to ensure university opportunities remained available:

*I know [my school at university] is like, it’s never explicitly homophobic—they have a pride club—but people have told me before, they go, “You might want to play down the whole gay thing when doing X because it might hurt your chances of doing Y.”*

#### Coming Out Concerns

As expected, navigation of LGB+ identities was often connected to the coming out process. Concerns about coming out were raised by six participants (38%). Many of these concerns related to coming out to family and friends. Phoebe (18, bisexual, White, woman), who was not out to her family, considered what might happen if she were to come out to her parents:

*They'd probably disown me. And then right now, since I'm still financially dependent on them, I don't know what would happen. If they were to disown me, like, what would happen to my college education, just paying for my college education? There's not having a place to go home to after, and just being really on my own. You know, I wouldn't be able to call them and have that supportive family figure. Instead, I’d have to rely on the people I have here, which isn't a bad thing because I already have a very strong community here. But you know, it's your family. And it's always been a safe space for me. And just not having that just kind of makes me anxious*.

Several participants described what led to concerns about coming out. Benjamin (21, gay, White man) explained why he is reticent to come out to part of his family:

*My grandparents on my dad's side have shared anti-LGBT stuff on Facebook. So, that's sort of given me these assumptions about how they would react, and that's kind of why I have delayed coming out to them for so long*.

Another participant, Jade (29, lesbian, biracial woman), reflected on her coming out experiences and the basis for related concerns:

*I think it was the stereotypes, of course, that kinda gave me fear in my mind that, you know, people will stereotype me or they would judge me. And I also struggled with friends. I was always self-conscious that I would lose lots of friends for, you know, finally coming out*.

Several participants also expressed concern with coming out to medical personnel, as Karine (25, lesbian, White woman) discussed:

*When I come out in that setting, this is actually, I would say, the setting I’m the least comfortable to come out with is with doctors, funnily enough*.

#### Coming Out Challenges

Specific challenges faced during coming out experiences were discussed by eight participants (50%). Javiera (26, bisexual, Latinx woman) described an early coming out experience from her adolescence:

*So, when I was in church, I came out to one of my really close friends there and she was like, “Oh, I can't be your friend anymore because that's just wrong. What you're doing is a sin.” … And like, I mean, I pulled away from the church in that instance. If you can't be friends with me because I decided to confide in you that I had a crush on a girl, I don't really want to be around that. So, I left the church, and I think that's the harshest response I’ve ever gotten*.

Loss of family, friends, and community connections are part of coming out for many LGB+ people—for some like Javiera, such losses are experienced, whereas others may fear such losses as they look ahead to the ongoing process of coming out.

#### Internalization of Sexual Identity Messages

Internalization of messages about LGB+ identities was discussed by five participants (31%), including Isaiah (32, gay, Black man), who stated:

*So, my partner talked to his mom last weekend about [our planned wedding]—we're not engaged, that’s too heteronormative, we do have a wedding date—and he said that he was shocked as to how much shame came up for him around that. And I think we both sort of looked back and we thought like, our discrimination and shame, the collective impact of it, is causing us not to do something, was slowing us down from doing the things in life that we want to do together*.

In this portion of his interview, Isaiah referenced internalization of broad-based negative messages ascribed to being gay that manifested as feelings of shame for both him and his partner. Patrick (23, gay, White man) named specific sources of the negative LGB+ messages he internalized:

*Patrick: I guess I should note, too, that I was raised Catholic. That's probably important to mention. So, I felt a lot of guilt with my identity and yeah, I mean, there's still a lot of homophobic, even broader sexist archetypes in society, and it still affects minorities in general in a lot of negative ways. Yeah, our perceptions and stereotypes do, for sure. But, specifically related to my identity, absolutely yeah, I have been negatively influenced, for sure. I always felt like I've been kind of taught to hate being gay for some reason*.

*Interviewer: Can you identify where those messages come from?*

*Patrick: Probably being raised in the South and being raised in the church. You know, I was never flat out told growing up that being gay wasn’t OK, but the way that gay people were talked about, it was never an extremely positive thing or wasn't ever really openly discussed. So, probably to have that taboo shroud around homosexuality definitely had a negative influence*.

As many participants referenced during their interviews, Patrick connected this response related to stigmatization of LGB+ identities to messages he received from his faith community and from living in the South. Yet regardless of challenges discussed, the interviews with many participants provided evidence of individual strengths and positivity in their navigation of an LGB+ identity.

#### Positive Coming Out Experiences

Positive coming out experiences were discussed by nine participants (56%), including this description of familial support received by Jade (29, lesbian, biracial woman):

*I think I got an easy family. They were already welcoming to it and very accepting to it. And just, I think they recognized the struggles that we go through day-to-day, and they've been a great support group for me. It's been much positive attitudes there*.

The importance of support from family when coming out cannot be understated. Relaying his mother’s response to his coming out as gay, Isaiah (32, gay, Black man) stated:

*She tried to act shocked, but I'm like, “Really honey? Really?” She had her own process with that—kind of jarred, worried, distressed. She told my father. He was very accepting of it—probably had less issues with it. You know, they were probably more worried about societal discrimination and how other people would treat me, rather than their own*.

#### Positive Sexual Identity Development

Development of a positive LGB+ identity was discussed by eight participants (50%). Alex (27, pansexual, White man) described how a positive coming out experience with his family fostered his positive identity:

*I would say that it probably helped my confidence and I've always been, I've had anxiety my whole life—diagnosed—and it definitely helped temper that because that was one fewer thing for me to worry about, especially internally. I didn’t have to deal with … “Oh god, I’m letting my family down by also being attracted to men.” So, I think it completely eliminated that worry in that aspect of coming to terms with myself because if they accepted me, why shouldn’t I?*

### Category 2: Social Acceptability of an LGB+ Identity

The category of social acceptability of LGB+ identities was characterized by participants’ descriptions of social acceptance of LGB+ people in the United States.

#### Faith and Geography

For 12 participants (75%), faith and geography figured into their perceptions of LGB+ acceptance. Such was the case for Jordan (25, pansexual, White woman), who recounted the following story about rural North Carolina:

*My partner is from a small town in [a different part of] North Carolina, very country sort of place, and the one out gay kid wound up being actually physically sought out for physical altercations. So, yes, I would say that that plays a big role—the South. The church—that’s a big one*.

Describing the potential for lack of acceptance due to sexual orientation by a faith community and the personal effects this can produce, Isaiah (32, gay, Black man), who grew up in the Catholic Church, stated:

*Joe Kort is an LGBT expert and a therapist, and he talks about not even having God to talk to when the church that you go to [doesn’t support you], and I remember times in my life where it's like, you question does your maker, does your creator really support you and can you even go to God because you’re worried that God is going to reject you? So, yeah, that’s some deep, painful shit*.

The role of faith in matters of social acceptance of sexual minorities was underscored by these comments from Jordan and Isaiah. For some, faith in the South was cited as a factor when describing their perspectives on LGB+ acceptance.

#### Pessimism About Social Acceptance

Pessimistic views regarding social acceptance of LGB+ identities were voiced by eight participants (50%). When asked to describe societal attitudes about LGB+ people in the United States, Phoebe (18, bisexual, White woman) responded:

*I'd say they greatly vary based on where you are. Like, here in the Bible Belt, it's predominantly negative or like, “Oh, being gay is a sin and you're wrong.” But in more progressive, in liberal places like New York or California, it's more accepted. But still I feel like there's kind of this negativity surrounding just like LGBT communities, because it's … I don't know, I feel like it seems it's frowned upon constantly*.

Similarly, Benjamin (21, gay, White man) connected his pessimism about LGB+ acceptance to his Southern roots:

*I think it very much has to do with where you come from. And where I am from … I am more pessimistic about acceptance of the LGB population just because that's kind of … I grew up with people, around people, that I thought weren't very accepting of it*.

Many participants simultaneously expressed pessimism and optimism about LGB+ acceptance during their interviews.

#### Optimism About Social Acceptance

Overall, more participants, 14 (88%), expressed optimism regarding LGB+ acceptance during their interviews. Parker (27, bisexual, Asian, gender nonbinary) articulated a bifurcated view on acceptance, noting both Southern status and the current political climate:

*I feel like there’s a lot of people who are supportive and a lot of people who are very unsupportive. This is the Bible Belt. With Trump as president, it has emboldened people to be more vocal*.

Although recognizing that work remains, Isaiah (32, gay, Black man) also expressed optimism about the country’s growing acceptance of the LGB+ population:

*But I think the needle has largely moved in the other direction of change and less stigma around it. And I think I can say that I've seen that in my own life. We have laws that kind of prove that, but we still have different faith communities and sects which have laws against adoption for LGBT parents. So, there's that. So, it's mixed, but I do think it's an improvement*.

In comparing disparate experiences in her home country and the United States, Priya (19, bisexual, Asian woman) noted that among her peers in North Carolina, the topic of sexuality is not taboo:

*And when I came here, I talked to people who are actually bisexual. And because [back home], no one talks about their sexuality, there's a lot of stigma around it, and no one's like, “Oh, yeah, I’m gay.” … But when I came here, I talked to people about their sexuality and they talked about being bisexual*.

For Karine (25, lesbian, White, woman), the social acceptability of an LGB+ identity in the United States has reached a point of becoming almost a nonissue:

*I think the main, most positive thing is that [it] is becoming not a big deal, like not a deal at all. It's like, “Oh, you're gay. OK. Cool.” That's like, oh, it's very, what’s the name … anticlimactic now, I think, to be gay*.

### Category 3: Expectation of LGB+ Stigma

The category of expectation of LGB+ stigma was characterized by participants’ expectations and worry regarding potential stigmatization due to their sexual minority identities. A majority of participants, 13 (81%), discussed personally expecting LGB+ stigma. Benjamin (21, gay, White man) detailed his expectation of friends’ negative reactions upon learning of his gay identity:

*I was very nervous to come out to friends, which I don't know why now … Any of my friends here [at university], it was fine, I knew they would be fine with it. But in high school, I just think my good friends from high school and back home, it just wasn't something that we ever talked about, or anyone really ever talked about. And so, I—for some reason, I think when I'm coming out, I assume the worst reaction possible and so that's kind of just what I assumed from my friends from home, just because I had no idea how they would feel about me. But I don't know why I ever worried about it*.

Though Benjamin stated his anxiety was for naught when coming out to peers that he “assume[s] the worst reaction possible” when coming out is indicative of substantial worry. Although many participants worried about how family and friends would respond to their LGB+ identities, others worried about being negatively judged by strangers. When visiting communities in more rural parts of North Carolina, Lara (50, lesbian, White woman) expressed her reasoning for worry and its negative effect:

*I think of it as maybe I stereotype and think there are gonna be lesser intelligent people or lesser educated people or less worldly, and so I'm expecting someone to just have a negative opinion of me and not be tolerant. And so, then I'm much more cautious and aware of who's around me and who's looking at me and stuff, and it just makes me uncomfortable most of the time that I'm there*.

Another source of expected stigma as indicated by participants was employment related. Jordan (25, pansexual, White woman), soon to be on the job market, discussed actions she has taken to circumvent potential negative reactions to her sexual orientation by potential employers:

*I'm graduating in May, and I have probably unnecessarily, but have done it anyways, scanned my Facebook for a lot of things, but primarily very polarizing views on pretty much anything LGBTQ. Anything indicating that I am one way or the other. On National Coming Out Day, like that post is deleted … I feel like I just don't want to take any chances, and despite the equal opportunity employment and everything else, the fact that Facebook is now something people are looking at when they are making hiring decisions, I feel like I need as clean of a slate, as wholesome of a slate as possible, and that's how I've acted on it*.

Like Jordan, Isaiah (32, gay, Black man) expressed worry about stigmatization from a specific source; his words echoed those of Karine, who discussed discomfort in coming out to medical professionals, except that Isaiah further voiced concern about the judgments they may render:

*I’m thinking about health care systems—it’s kind of an uncomfortable space to be in with new providers for me. Talking about sex practices and then wondering, what are they assuming about me?*

Unlike statements from previous participants that addressed sexual identity stigmatization from specific sources, Priya (19, bisexual, Asian woman) described a broad-based anxiety about potential discrimination:

*I do worry about it, and I worry about getting some sort of backlash that I don't like. I feel like I'm in a state right now that I care a lot about what people think about me for some reason, and I want to get out of that. But at this point, I am not, and so I do worry about people just discriminating against me or being violent or hurting me*.

Similarly, Parker (27, bisexual, Asian, gender nonbinary) expressed a generalized sense of anxiety about the potential for stigmatization due to their sexual minority status, but their worry was primarily focused on the well-being of others in the community:

*It is concerning. I guess because there’s so many layers to discrimination, I don’t think I would be targeted compared to those who look more queer than I do. It makes me scared for them. Will something happen to them? I feel like that could happen to me. It definitely worries me*.

### Category 4: Interpersonal Discrimination and Harassment

The category of interpersonal discrimination and harassment was characterized by personal experiences with LGB+ discrimination (e.g., verbal epithets and physical violence), as well as questioning whether certain experiences were discriminatory and motivated by sexual minority identity.

#### Personal Experiences

Nearly all participants, 15 (94%), recounted personal experiences with LGB+ discrimination. During his interview, Kevin (48, gay, White man) described an early experience in his career in a Southern state:

*I remember in grad school there was a position open at [my university] and I wanted the job. I was the intern already for 2 years and they were hell-bent and determined that I wasn't gonna even be interviewed for it. A friend of mine who worked there said that she thought it was because I was gay*.

More than a decade later, in 2010, Kevin was arrested in a mid-sized North Carolina city and described the reason for his arrest and its impact in the following manner:

*Kevin: My experience with it was as a part of a sting operation where they arrested me for blowing somebody and I didn't even do it. But when they charged me, it went on my record as “crimes against nature.”*

*Interviewer: How would you say that experience affected you?*

*Kevin: I lost a job, a career—it impacted me big time*.

Like Kevin, Phillip’s (46, gay, White man) life intersected with the criminal justice system; having previously been in prison in North Carolina, he recounted an escalating experience with another person who was incarcerated that involved verbal epithets, threats, and violence:

*And he jumped up and he started going, “Faggot! Faggot! Faggot!” Over and over at me. And I said, “Would you please stop calling me that. Please stop calling me that. Please stop.” And I kept saying that and he kept calling me faggot over and over and he kept getting louder and louder. I tried to get up and leave and he blocked me into the bunks, and I couldn't get out. And he kept doing it, kept doing it, kept doing it, kept telling me he's gonna kill me. This went on and on, and I kept trying to leave, and every time I'm trying to leave, he gets like this [participant indicates other incarcerated individual was getting in his face] and he tells me he’s gonna hit me. He finally hit me, and I fell*.

Other participants described forms of sexual identity discrimination, including that which occurs in families. While discussing coming out to his mother, Benjamin (21, gay, White man) recounted the following experience:

*At a certain point, I just like broke down and I came out to her in that moment on the phone and she had the worst reaction of anyone that I've come out to, and I think in perspective, it was terrible for me at the moment. I'm trying to not say my experiences are worse, I mean, definitely not as worse as a lot of people—as bad as a lot of people's experiences coming out—but it was pretty bad. And she just started bawling and telling me that it wasn't true, that I whatever, that I needed to talk to our priest. That my brother had just had a kid a month before that and … I was going to be the godfather—I’m still the godfather of his kid—and telling me he's gonna not let me be the godfather of his kid anymore. And even, at times, insinuated going to some kind of therapy to try and change it. And it was extremely, like, really, really, really upsetting*.

Patrick (23, gay, White man) was one of two participants to discuss the bullying he experienced:

*I started to be bullied probably around like 12 or 13, before I even knew I was gay. … I never even questioned my sexuality and was just brought up thinking I was straight. So, to hear other kids say that I was gay, you know, and telling me, “You are gay,” was a really traumatic experience, and I definitely had some pretty awful things happen to me*.

Like Phillip’s experience in the criminal justice system, other participants recounted the various slurs and violent remarks aimed at them by others. Karine’s (25, lesbian, White woman) comments illustrate the sexualized verbal epithets that sexual minority women often endure:

*It's always men—I've never had any, no female has ever called me a name. But with men, it's either sexual, so you know, like, “Oh, y'all are so hot. Let me join,” or whatever, those kind of like sexualizing us and wanting to take a part in that. Or it's like “dyke,” or whatever that is – and especially in cars, ‘cause people when they're in the car, and when you walk on the street or whatever, they feel like they won't get confronted. So, a lot of time, it's people shouting from cars, screaming from cars, motorcycle, whatever, and then driving away*.

Parker (27, bisexual, Asian, gender nonbinary) described experiencing two types of verbal epithets—violent comments posted online and slurs from family members:

*Most discrimination I experience occurs on social media – comments like, “disgusting,” “You should die,” or “You should be executed.” I’ve also been called a “dyke” a couple of times by my brothers*.

#### Questioning Experiences as Discriminatory

When discussing sexual minority-related discrimination, six participants (38%) questioned whether potentially discriminatory experiences were related to their sexual orientation. Lara (50, lesbian, White woman) wondered about job opportunities for which she was not hired:

*I've applied for and interviewed for jobs that I thought I was perfectly qualified for and then I didn’t get the job. I don’t think it was because of my appearance and being a lesbian, but I'm sure it was related to the job itself. But sometimes I've wondered is it because of that. You know, like who am I up against? If I'm the best qualified out of the whole group, is it because they don't want someone who looks like me in that particular field or whatever?*

Whereas Lara questioned if such lost opportunities were attributable to her identity as a lesbian or her gender presentation, Casey (20, lesbian, biracial woman) queried broadly about her intersecting marginalized identities and whether she experienced discrimination as a result:

*But I don’t think anything’s ever really been taken away, like any major opportunities, because of it. Who knows, you know? I’m a triple minority—I got woman, I got biracial, and I got gay. So, who knows what is happening, what could happen, and what's not happening? I don’t know*.

### Category 5: Structural Stigma

The category of structural stigma was characterized by participants’ discussion of legal and policy issues that affect them as LGB+ individuals, including what they think about the status of current LGB+ laws or legal decisions.

#### Feeling Excluded, Marginalized, or Not Protected

During their interviews, 10 participants (63%) discussed feeling excluded, marginalized, or not protected by proposed or existing LGB+ laws and institutional policies. Referencing North Carolina’s constitutional amendment approved by voters in 2012 that restricted marriage to one man and one woman that was later declared unconstitutional, Robin (50, lesbian, White woman) stated:

*I almost moved out of North Carolina when Amendment One came. … If I’m going to pay taxes, I’m sure as hell not going to be written out of the Constitution. I’m sorry, they will not get my money*.

Expressing frustration at the lack of political movement to enact protections for sexual minorities at the federal level, Alex (27, pansexual, White man) pivoted to discussion of state laws:

*I don't expect specific laws to be made. I'm almost beyond hope for protection, anti-discrimination protection, at least on the federal level. I can see it happening in various states if it hasn't already in various states. But you even have places where people are prohibited from making—North Carolina is an example of that, and I can't believe I didn't think of HB 2. Oh my god. Oh my goodness. I can't believe I didn’t think of that. But part of the, one of the conditions for HB 2 was local governments can't make any rules regarding that, and that's an overstep*.

Though Alex had pinned his hopes on legislative action to protect sexual minorities at the state level, he was reminded of HB 2 (i.e., the Public Facilities Privacy and Security Act) in North Carolina and the legal action taken in the state to end this law that at the time of his interview, continued to exclude LGB+ people from local anti-discrimination protections. For Isaiah (32, gay, Black man), discussion of legal issues included the effects of HB 2 and challenges related to adoption for prospective LGB+ parents:

*I’m thinking about HB 2, in short, you know, talking about LGB folks, I do think it kind of is a ripple effect for the whole community and people that we're all aware of. And then it has you in this kind of hypervigilant state of “What's next?” I think adoption laws have been an issue. Thinking about starting families and what the choices are around that*.

Though North Carolina’s HB 2 primarily targeted the transgender community, it is evident that for some LGB+ participants, it continues to have an effect, especially given the limits of its replacement bill on anti-discrimination protections in the state. The marginalization participants felt as a result of anti-LGB+ laws or laws that exclude the LGB+ population was also felt by participants faced with anti-LGB+ institutional policies. Kevin (48, gay, White man), a middle school teacher in the North Carolina public school system, described the communication of an unwritten policy:

*Kevin: The last couple of years, I've had a few students where I knew they're gay, they tell me they're gay, but yet I'm not allowed to say anything back to them*.

*Interviewer: Is that a written policy or is that a verbally communicated, unwritten policy?*

*Kevin: Verbally communicated and unwritten. I don't remember seeing anything written*.

*Interviewer: What did you think when you were told that you shouldn't or couldn't tell them?*

*Kevin: I thought it was ridiculous, for one. It doesn't really make you feel good*.

Further emphasizing the notion that laws and policies do not have to be enacted to have an impact, Casey (20, lesbian, biracial woman) discussed the effect of anti-LGB+ legislation:

*Whether or not these laws get passed … the fact that they’re at least in question kind of validates other people's maybe bigoted beliefs. You know, if they’re like, “Well, if this guy can get this far with this anti-gay bill, then maybe I think it's OK to be homophobic.” So, even if it doesn’t directly affect you or if it doesn’t actually come to fruition, it’s definitely like there's an impact*.

Casey’s description of the legitimacy conferred upon “bigoted beliefs” by either proposed or enacted anti-LGB+ laws is a powerful statement on how such governmental actions further stigmatize LGB+ identities.

#### Precarious Nature of Protections

Additionally, eight participants (50%) discussed the “precarious” nature of existing LGB+ civil rights and protections. Lara (50, lesbian, White woman) stated:

*I mean, things can still be precarious as far as like marriage equality. I mean, that's kind of law of the land now, but you never know with particular politicians and government that's in place … it’s almost like we still have to be guarded*.

Casey (20, lesbian, biracial woman) reiterated the notion that people with LGB+ identities must remain attentive to political processes to safeguard existing rights:

*Straight people, it’s their privilege to be like, “Oh, I’m not really a political person.” But I feel some of us, we’re not able to say the same because if we’re ignorant of politics, who knows what rights are going to be taken away. We always have to be kind of conscious of it*.

#### Feelings of Inclusion or Protection

In addition to the stigmatization resulting from laws and policies, six participants (38%) described feelings of inclusion or protection from such structures. Referencing institutional statements of LGB+ inclusivity, Robin (50, lesbian, White woman) stated:

*You know, every time I hear about one of those, I get a little buzz. It makes me a little happier. The more inclusionary statements, and I think it’s the same … it really is, it’s exactly the same spark that I get when I hear the religious inclusivity statements that school districts and stuff like that are putting out, you know?*

Benjamin (21, gay, White man), a soon-to-be college graduate, recently secured a job with a company that actively included and supported LGB+ employees:

*I know the company I’ll be working for next year is, or after graduation, they have like a huge, a really big campaign around inclusion and diversity. They have pride groups that do a lot of stuff together and professional networks. And so that was kind of one of the motivations that I was looking for in jobs, was finding a place that made a big effort*.

Having the right to marry and marrying her wife, Jade (29, lesbian, biracial woman) discussed the positive, personal effects of marriage on her lesbian identity:

*It makes me feel accepted, again. It just makes me feel like my sexuality, my lifestyle—it's just a regular old lifestyle, it’s just with another woman. I felt like I'm able to, I want to be the success story behind a marriage with a woman—two women being married. And so, for me I just want to live a regular life, you know. And not really care about who my partner is, but having that partner with me and just live my regular life—create a family*.

### Category 6: Relationship With the LGBTQ Community

The category of relationship with the LGBTQ community was characterized by participants’ descriptions of personal relationships with the LGBTQ community. In addition to positive experiences with the LGBTQ community, some participants felt stigmatized by the community due to their identities as bisexual or pansexual or intersectional identities inclusive of their race or ethnicity. For some, this stigmatization by the LGBTQ community contributed to feelings of disconnection.

#### Conflicted Relationship With the Community

Six participants (38%) described conflictual relationships with the LGBTQ community. Jordan (25, pansexual, White woman) described exclusionary sentiments among some in the community:

*“Are you queer enough?” Like, if someone who identifies strictly as a lesbian is dating someone who’s bi, it becomes “Oh, well, she's not really you know, technically a lesbian, she's halfsies,” you know. So that's, that's definitely there*.

Like Jordan, Alex (27, pansexual, White man) brought up the erasure of bisexual and pansexual identities in the LGBTQ community:

*I believe in the LGB community that bi [and] pan erasure is a thing. It's either you're gay or you’re not, you’re either lesbian or you’re not. And that's not a strictly enforced thing, there's just a lot less talk about bi and pan people*.

Alex also described his struggle with feeling as though he does not have a clear place in the LGBTQ community as a result of his various identities:

*As a man who is attracted to women as well as other men and anywhere on the spectrum … I personally feel like it's hard for me to find a place because I am so close to heteronormative. I'm currently in an opposite-sex relationship. I am a cisgender White man, but I do feel, at minimum, I feel a kinship to other LGBT people. And a lot of my friend group is. But with the community as an organized community, I have a hard time feeling right*.

In these quotations from Jordan and Alex, the perception of not being or feeling “queer enough” emerged. For Phoebe (18, bisexual, White woman), who is not out to her family, the personal effect of bisexual stigma from the LGBTQ community gives her pause to consider further connection with the community and adds to the stigmatization she feels from her home community:

*It definitely has a little bit just hindered me, just like, “Do I really want to join a community that doesn’t fully recognize bisexual people?” That has come across my mind. And that I feel like is something that's keeping me from joining that broader community. But I guess it depends on, you know, where that community is. But in general, across the board I've seen that be a trend. So, I’m just like, OK, I don’t really know if I want to be part of the community that degrades me further for being bisexual. Like, I already have other people doing that, you know, back at home, so I don't need this large community doing that, too*.

In addition to stigmatized polysexual identities (e.g., bisexuality and pansexuality), the data revealed other forms of stigmatization in the LGBTQ community. Isaiah (32), a gay Black man, discussed stigma resulting from the idealized gay man in the LGBTQ community—an image different than his intersecting identities:

*It can be, there can be some sort of shame and some toxic messages of what we think it means to be a gay man and so having to sort of liberate myself from, you know, you have to look like an Instagram model and you have to be White in order to be desired is something that is continuous work even today*.

#### Positive Relationship With the Community

For eight participants (50%), the LGBTQ community connotes positivity; these participants expressed either a connection to the LGBTQ community or a desire to deepen such connections. Many participants, including Lara (50, lesbian, White woman), discussed the importance of attending Pride events:

*It's my people—and it’s not just all gay people who go to that certainly. It’s supporters, straight supporters, it’s just a mix of people that show up for that. It’s that sense of here are hundreds, if not a couple thousand of my people … A place where I could walk around, hold hands with someone, just be completely comfortable*.

Similar to Lara, Jade’s (29, lesbian, biracial woman) comments highlighted her strong sense of LGBTQ community affiliation and what that connection provides her:

*It just kind of helps me feel prideful in my sexuality. And it also, because [there is] such a strong fight for the LGBT community, so to be connected—it kind of feels like we’re all working together and being able to kind of fight for our rights and kind of feel like we’re the same*.

## Discussion

Sixteen LGB+ North Carolinians discussed their experiences to help answer the question: What are the experiences related to sexual identity stigma among self-identified LGB+ adults in the present-day United States South? As the narratives detail, participants’ perspectives varied regarding experiences with sexual identity stigmatization in the South. Regardless of their characterization of the changing American landscape for sexual minority populations, each participant reported experiencing sexual identity stigma.

The first five thematic categories align with conceptualizations of minority stress (Meyer, 2003) and sexual identity stigma (Herek, 2007). In accordance with minority stress theory, proximal stressors emerge from the first three categories (i.e., navigating an LGB+ identity; social acceptability of an LGB+ identity; and expectation of LGB+ stigma), while distal stressors emerge from the fourth category (i.e., interpersonal discrimination and harassment). In relation to the sexual identity stigma framework, individual manifestations of stigma are present in the first four categories (i.e., navigating an LGB+ identity; social acceptability of an LGB+ identity; expectation of LGB+ stigma; and interpersonal discrimination and harassment), with structural manifestations of stigma present in the fifth category (i.e., structural stigma).

Experiences with sexual identity stigma were frequently connected to participants’ navigation of their sexual minority identity. For many participants, stigmatization of LGB+ identities contributed to concerns about their coming out, the challenges they experienced after coming out, concealment of identity, and self-censorship of identity. The often-protective strategies of identity concealment and censorship are common among people with LGB+ identities. Described by Meyer (2003) as minority stressors, identity concealment and censorship potentially contribute to negative health outcomes (Meyer, 2003; Hatzenbuehler, 2009). Additionally, fears of being stereotyped and judged, and potential loss of family and friends were indicated by some participants as antecedents inhibiting their coming out. Potential loss of familial ties and financial support that may result from coming out was a fear that surfaced and is a concern echoed by other LGB+ people as they consider the possible effects of coming out on their lives (Jadwin-Cakmak et al., 2015; Chester et al., 2016; Goldbach and Gibbs, 2017). Identified concerns about coming out were also linked to discomfort in coming out to medical professionals—a concern noted in the literature (Barbara et al., 2001; Aleshire et al., 2019) that has implications for the health of sexual minorities.

Recounting navigation of LGB+ identities, participants frequently discussed the negative impact of geography and faith. Conservative political and religious ideologies were bound to participants’ Southern homes (and for most, their upbringings), resulting in barriers to positive sexual minority identity navigation. Participants highlighted negative societal messaging of sexual minority identities, often linked to faith and life in the South and negative self-perceptions, providing evidence of internalized stigma—a frequent subject in the extant literature often linked to negative mental health among sexual minorities (Newcomb and Mustanski, 2010; Fredriksen-Goldsen et al., 2013). Despite experiencing difficulties navigating their LGB+ identities, many participants discussed the role of positive coming out experiences and identity development, underscoring the growing social acceptance of LGB+ identities. Positive findings, such as these, underscore the range of stigma-related experiences among participants and indicate resources that may mitigate sexual identity stigma.

Most participants expressed a mix of pessimism and optimism when asked to describe their views on LGB+ acceptance in the United States. Stigmatization of LGB+ identities, often linked to a perceived strict Christianity (e.g., the Bible Belt) and Southern political conservativism, fostered pessimism among many participants regarding social acceptance of sexual minorities. Pessimistic views on LGB+ social acceptance were seemingly connected to expectations of rejection or stigmatization included in both minority stress theory (Meyer, 2003) and the sexual stigma framework (Herek, 2007). However, consistent with research indicating growing acceptance, nearly all participants voiced optimism for the improving social climate for LGB+ people in the United States, with one participant expressing agreement with scholars, such as Savin-Williams (2005), McCormack (2012), and Kirchick (2019), that stigma has significantly decreased.

Overwhelmingly, participants expected stigmatization due to their sexual minority identities. For many, this expectation led to anxiety. Expected stigma, as previously indicated, is consistent with the frameworks of Meyer (2003) and Herek (2007). Participants expected sexual identity stigmatization to come from various sources—from generalized conceptions to specific notions—including potential employers, people who live outside the LGB+-friendly communities where participants live, and friends and family. Additionally, the form that expected sexual identity stigmatization might take varied, ranging from worry about negative judgement to the potential for experiencing violence. Expectation of stigma that leads sexual minorities to be hypervigilant is often discussed in the literature as a minority stressor (Meyer, 2003) contributing to negative health outcomes in the LGB+ population (Lewis et al., 2003).

With near unanimity, participants experienced sexual identity-based discrimination. Similar to the various types of expected stigmatization engendering worry among participants, discriminatory experiences highlighted by participants were wideranging, including job loss or lack of consideration for employment, legal discrimination, violence, bullying, and verbal epithets and threats from both online and real-life sources, such as family members. In some instances, faith and Southern geography played roles in discrimination. Experienced discrimination is a primary form of sexual identity stigma described in both the theoretical (Meyer, 2003; Herek, 2007) and empirical literature (Herek, 2009; Tilcsik, 2011; Pew Research Center, 2013; Gates and Viggiani, 2014). That two participants (13%) in this study had significant engagement with the North Carolina criminal justice system is likely not a coincidence. Recent research indicated that LGB+ people are overrepresented in the American criminal justice system (Meyer et al., 2017). Further, that one participant was charged with solicitation of “crimes against nature” in a local North Carolina police sting operation highlights existing structural stigma and views regarding same-sex sexual relations, as such anti-sodomy laws were declared unconstitutional by the Supreme Court’s 2003 Lawrence v. Texas decision (Christensen, 2014), yet remain in 12 states (eight of which are Southern; Villareal, 2019). Particularly harmful forms of discrimination experienced by participants were family-based discrimination (see Ryan et al., 2009; Hall, 2018), bullying experienced by LGB+ youth or youth perceived to be LGB+ (see Varjas et al., 2008), and physical violence (see McKay et al., 2019). Finally, for some participants, it was unclear whether certain experiences were related to their sexual minority identities, particularly for those cognizant of their intersecting identities.

Despite numerous participants stating they did not follow politics closely or were not versed in the specifics of LGB+ laws, many articulated the personal and societal effects of structural stigma. Whether citing proposed or enacted laws, unwritten or written policies, or the lack of LGB+ legal protections, the exclusionary impact of anti-LGB+ (or nonexistent) laws and policies was felt by participants. For many in this North Carolina sample, state-level anti-LGB+ laws were frequently discussed and viewed as part of a larger, harsher form of sexual identity-based structural stigma. Particularly potent was recognition of the role of anti-LGB+ laws in sustaining sexual identity stigma, an important aspect of the theories underpinning stigma (Link and Phelan, 2001; Herek, 2007) that in recent years have gained greater empirical attention (Hatzenbuehler et al., 2009, 2012). Additionally, participants often assessed existing sexual minority rights and protections as precarious, expressing the need for personal and community vigilance to ensure such laws remain in place. Participants also discussed the positive impact of pro-LGB+ laws and policies, emphasizing how such structures validate their personal LGB+ identities.

Departing from the frameworks of Meyer (2003) and Herek (2007) is the final thematic category to emerge from the interview data—relationship with the LGBTQ community. Theoretically, sexual stigma is conceived as derivative of heterosexism and its denigration of LGB+ identities. Given more than one third of participants reported feeling stigmatized by the LGBTQ community, this “intracommunity stigma” represents a needed addition to existing theories of minority stress and sexual identity stigma. Consistent with findings from other studies (Hequembourg and Brallier, 2009; Roberts et al., 2018), participants with polysexual identities expressed sexual identity stigmatization from an LGBTQ community dominated by lesbians and gay men. This devaluation and erasure of polysexual identities by the LGBTQ community are linked to decreased mental health (Friedman et al., 2014; Flanders et al., 2017). Further, participants described the unattainable standards of what it means to be a part of the LGBTQ community, particularly for LGB+ people of color, as another way they experience intracommunity stigma. Because connections with the LGBTQ community are linked to greater levels of social and psychological well-being for sexual minorities (Kertzner et al., 2009), it is important that all members of the community have a safe, supportive, and nonstigmatizing place in the community. For many participants who expressed a positive relationship with the community, this is the personal experience described as they voice a sense of community, and feeling comforted, supported, and prideful in their sexual identity as a result of their LGBTQ community connections.

### Implications for Policy and Practice

The current findings have important implications for intervention efforts related to stigma experienced by the LGB+ community. Given the pervasiveness of stigma, mental health practitioners should consider interventions across the bioecological spectrum to address immediate client needs, encourage growth in LGB+ social acceptance, and reduce sexual identity stigmatization. To support LGB+ clients, practitioners should assess community needs when possible to determine intervention interest and acceptability; potential interventions to support clients as they cope with the various effects of stigmatization include individual counseling, social network-building programs, and support with coming out. The development and implementation of training programs for professionals (e.g., teachers, faith-based leaders, and medical professionals) provide opportunities to both increase social acceptance of sexual minorities and effect change in societal institutions. An example of such a training program is the University of Louisville’s LGBT Health Certificate Program that promotes medical students’ LGB+ health knowledge and improvement of attitudes toward sexual minorities (Sawning et al., 2017). Further, training programs aimed at health professionals may also foster increased comfort of LGB+ patients with medical practitioners. Structural change should be a practitioner focus, including advocacy for policies and legal protections inclusive of LGB+ people, and elimination of discriminatory and exclusionary policies and laws. Relatedly, structural interventions should include community-based programs aimed at building political advocacy capacities of the LGBTQ community. Finally, through awareness campaigns, educational programming, and intentional outreach, the LGBTQ community should address its stigmatization of polysexual identities and bodies that do not conform to the often unattainable, idealized standards of what it means to be LGB+. As practitioners intervene to address sexual identity stigma, consideration should be given to expansion of services in geographic regions and communities that have historically been excluded from service provision. Additionally, alternative methods of service delivery, including use of mobile and non-mobile communication technologies, should be explored as means to connect with hard-to-reach LGB+ populations (e.g., rural residents, individuals with concealed sexual identities; McInroy et al., 2019).

### Limitations and Future Directions

The findings of the current study should be considered alongside the following limitations. First, consistent with qualitative research, findings only reflect the experiences of study participants, and caution should be taken in generalizing to all LGB+ North Carolinians or Southerners. Second, use of purposive sampling resulted in study participants who were likely to be more comfortable with their sexual identity and discussing related experiences, as well as participants who were more likely to have LGBTQ community connections and thus, may have experiences and perspectives that differ from the broader LGB+ population. Third, recruitment methods resulted in a mostly White, cisgender sample, further limiting the perspectives in the interview data. Finally, due to the nature of stigma—with its explicit and implicit aspects—it stands to reason that certain experiences of sexual identity stigma went unnoticed or unacknowledged by participants.

Researchers should continue exploring experiences of sexual identity stigma among sexual minorities in the Southern United States, expanding to include greater geographic diversity and diversity in sexual minority populations. Increased inclusion of diverse queer Southern voices (e.g., sexual minority identities, racial and ethnic identities, immigrants, people with disabilities, levels of outness, and faith traditions) in future research will increase understanding of how intersecting identities affect experiences of sexual identity stigma. Additionally, sexual identity stigma research should expand in other regions of the United States (i.e., Midwest and Mountain West) often neglected in the LGB+ stigma literature. Comparisons of sexual identity stigma between states or regions of the country will aid understanding of this phenomenon. Changes in the sociopolitical landscape for sexual minorities at federal, state, and local levels provide opportunities for examination of how such changes affect sexual identity stigma. Relatedly, the collection of sexual identity data in population health surveys will allow for examination of how structural changes (e.g., laws and policies) affect sexual minorities over time. Exploratory study of sexual minorities’ experiences with intracommunity stigma is needed to further understand the scope and impact of this phenomenon. Beyond further research with cisgender sexual minorities, researchers should focus on the study of gender identity stigma experienced by transgender and gender-diverse populations in the United States South, including individuals with intersecting transgender and gender-diverse and sexual minority identities.

## Conclusion

In an American sociopolitical environment improving for many sexual minorities, LGB+ Southerners continue to face sexual identity stigma. Regardless of how participants perceived the changing American climate for sexual minorities, sexual identity stigma was a unifying experience among study participants. Though exploratory in nature, much of this study’s data align with existing constructs of minority stress (Meyer, 2003) and sexual identity stigma (Herek, 2007). An exception to this theoretical alignment was intracommunity stigma as an aspect of participants’ relationships with the LGBTQ community. For many in this study, their experiences with sexual identity stigma were tied to harsher forms of stigma in the South (e.g., structural stigma) in which religion and a conservative political system play significant roles. The participants’ lived experiences stand in contrast to arguments that sexual identity stigma in the United States is no longer a significant factor in LGB+ lives.

## Data Availability Statement

The raw data supporting the conclusions of this article will be made available by the authors, without undue reservation.

## Ethics Statement

This study involving human participants was reviewed and approved by the Institutional Review Board of the University of North Carolina at Chapel Hill. The participants provided their written informed consent to participate in this study.

## Author Contributions

All authors contributed to the conception and design of the study. JF conducted the data collection, performed the content analysis, and wrote the first draft of the manuscript. WH and PL supervised the process of data collection. WH supervised the content analysis. WH, JG, and PL contributed to manuscript revision. All authors contributed to the article and approved the submitted version.

## Funding

Research reported in this article was supported by the National Institute of Minority Health and Health Disparities of the National Institutes of Health under award number R21MD012687. Other support was provided by the School of Social Work at the University of North Carolina at Chapel Hill under WH’s research funds.

## Conflict of Interest

The authors declare that the research was conducted in the absence of any commercial or financial relationships that could be construed as a potential conflict of interest.

## Publisher’s Note

All claims expressed in this article are solely those of the authors and do not necessarily represent those of their affiliated organizations, or those of the publisher, the editors and the reviewers. Any product that may be evaluated in this article, or claim that may be made by its manufacturer, is not guaranteed or endorsed by the publisher.
